# Cross-Cultural Adaptation and Clinical Validation of the Mini Sarcopenia Risk Assessment Questionnaire in Community-Dwelling Spanish Older Adults

**DOI:** 10.3390/diagnostics14192123

**Published:** 2024-09-25

**Authors:** Inés Moreno-Sánchez, Agustín Aibar-Almazán, María del Carmen Carcelén-Fraile, Ana Belén Parra-Díaz, Indalecio Sánchez-Montesinos García, Marcelina Sánchez-Alcalá, Javier Cano-Sánchez, Fidel Hita-Contreras

**Affiliations:** 1 Department of Health Sciences, Faculty of Health Sciences, University of Jaén, 23071 Jaén, Spain; 2 Department of Education and Psychology, Faculty of Social Sciences, University of Atlántico Medio, 35017 Las Palmas de Gran Canaria, Spain; 3 Department of Human Anatomy and Embryology, University of Granada, 18016 Granada, Spain

**Keywords:** sarcopenia, screening, reliability, validation

## Abstract

(1) Background: The aim was to analyze the reliability and validity of the Spanish version of the Mini Sarcopenia Risk Assessment (MSRA) in older adults. (2) Methods: A total of 136 participants (72.24 ± 5.21 years, 68.38% women) took part in the study. The MSRA includes two questionnaires with seven (MSRA-7) and five items (MSRA-5). First, reliability (inter-rater and test–retest) of the Spanish MSRA was studied, and then the total scores were compared with the presence of sarcopenia according to three different diagnostic criteria and with other parameters related to sarcopenia (clinical validation). (3) Results: The analysis showed excellent inter-rater and test–retest reliability. As for the clinical validation, and regardless of the criteria, both questionnaires had a high sensitivity (81.82–88.89% for the MSRA-5 and 90.91–94.44% for the MSRA-7), while the MSRA-5 showed a better specificity (32.00–33.90%) than the MSRA-7 (20.80–22.88%). Predictive positive values ranged from 9.57–17.02% (MSRA-5) and 9.17–15.54% (MSRA-7), while predictive negative values were high for both the MSRA-5 (95.24%) and the MSRA-7 (96.30–96.43%). The accuracy was better for the MSRA-5 (36.03–41.18%) than the MSRA-7 (26.47–32.35%), as well as the area under the curve (0.67–0.76 vs. 0.65–0.73, respectively). Higher MSRA-5 and MSRA-7 total scores significantly correlated with greater muscle strength, quantity and gait speed. (4) Conclusions: The adaptation of the Spanish MSRA questionnaires was successfully performed, and they are reliable and clinically valid tools for assessing sarcopenia.

## 1. Introduction

Sarcopenia has been defined as a progressive and generalized disorder of skeletal muscle that is linked to higher odds of many adverse health-related outcomes, such as physical disability or increased morbidity and mortality [1]. 

Originally, sarcopenia diagnosis was based only on muscle mass [2], but in the last years, different study groups have included muscle strength and physical performance to the diagnostic operational criteria. In 2010, the European Working Group on Sarcopenia in Older People (EWGSOP1) met for the first time [3], and in early 2018, the group met again (EWGSOP2) to update the definition of sarcopenia [1]. In addition, in 2011, the International Working Group on Sarcopenia (IWGS) provided a consensus definition of sarcopenia [4], and in 2014, the Asian Working Group for Sarcopenia (AWGS) proposed a diagnostic algorithm based on Asian data in 2014 [5], which was also updated in 2019 (AWGS-2019) [6]. In 2016, sarcopenia was recognized as an independent condition by the International Classification of Diseases, Tenth Revision, Clinical Modification (code M62.84) [7].

Sarcopenia has been associated to many health-related poor outcomes, such as cardiovascular disease [8], mild cognitive impairment, Alzheimer’s disease and other forms of dementia [9], inflammatory bowel disease [10], poor prognostic factor for patients with cancer [11,12,13], poor health-related quality of life [14] and increased mortality [15]. However, sarcopenia is important not only in terms of health but also from an economic, social and personal point of view [16].

For this reason, the screening of this condition is of great importance. With this purpose, some tools have been developed, such as the SARC-F (strength, assistance in walking, rise from a chair, climb stairs and falls) questionnaire [17], a widely used five-item scale with a very high specificity but poor screening sensitivity performance [18]. The Mini Sarcopenia Risk Assessment (MSRA) questionnaire is a reliable and validated instrument for sarcopenia risk screening in older adults, developed by Rossi et al. in 2017 [19], whose sensitivity is higher than that of the SARC-F, although its specificity is lower [20]. This tool has a full version with seven items (MSRA-7) and short one with five items (MSRA-5), which have proven to have good sensitivity for the identification of the risk of sarcopenia. To date, the MSRA questionnaires have been cross-culturally adapted to and validated in several languages [21,22,23,24,25], but to the best of our knowledge, the validation of the Spanish version of the MSRA questionnaires has not yet been performed. 

Therefore, the objective of the present study is to carry out the translation and cross-cultural adaptation of the Spanish versions of the MSRA questionnaires and determine their reliability and clinical validity in Spanish older adults who live in the community. We hypothesized that the Spanish MSRA questionnaires are reliable and clinically valid tools for screening sarcopenia.

## 2. Materials and Methods

### 2.1. Study Design and Participants

**Figure 1:**
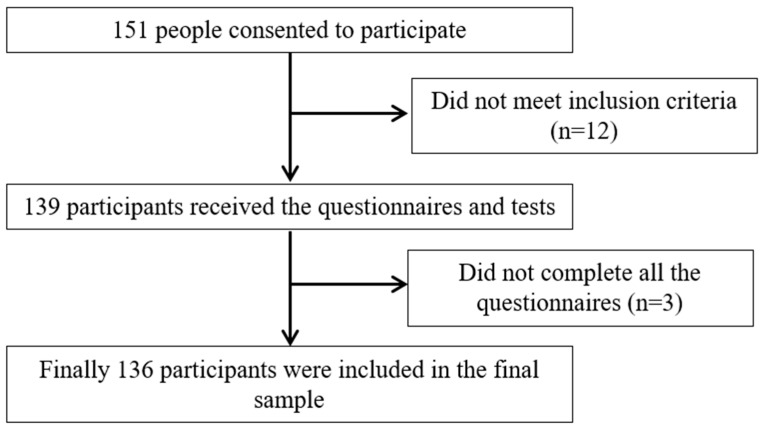
Flow diagram of the study participants.

A cross-sectional observational study was carried out in September and November 2023. The study initially reached out to 151 older adult volunteers from two day care centers in Jaén (Spain), and, ultimately, 136 individuals participated (Figure 1). This sample size is appropriate following the psychometric recommendations provided by Kline [26]. Participants were eligible if they were 65 years or older, native Spanish speakers, capable of walking independently or with assistance in safe conditions, agreed to complete the questionnaires and understood the study’s objectives. Exclusion criteria included being bedridden, having contraindications for bioimpedance (metal implants, cardiac pacemaker, etc.), suffering from chronic or severe medical conditions that could affect their responses and not providing their willingness to participate in this study. Informed consent was obtained in writing from each participant before the beginning of the study, which was approved by the Ethical Committee of Clinical Research of the University Hospital of Santa María del Rosell (Murcia, Spain) and was carried out following the Declaration of Helsinki, good clinical practices, and all applicable laws and regulations.

### 2.2. Procedure

The translation and cultural adaptation of the Spanish version of the MSRA questionnaire was carried out according to the two phases described by the World Health Organization (WHO) methodology for translating and adapting health questionnaires across different cultures [27]. We received authorization from Dr. Andrea Rossi, one of the co-authors of the original MSRA, to perform the validation and cross-cultural adaptation of the Spanish version of the MSRA-5 and MSRA-7 questionnaires. In the initial phase, the translation and cultural adaptation of the MSRA-7 and MSRA-5 questionnaires into Spanish were conducted. First, a consensus preliminary Spanish version was obtained by two bilingual experts together with clinical professionals who were familiar with this topic. This version was completed by 10 subjects (5 men and 5 women) to ensure that the questions and instructions were clear and comprehensible. Subsequently, the back-translation process to the original language (English) was performed, and both versions were compared. Two independent experts assessed inter-rater reliability in a sample of 20 participants (10 men and 10 women). Test–retest reliability was assessed by two independent researchers in 25 participants who completed the questionnaire again two weeks later. For the second phase, we conducted the clinical validation of the Spanish MSRA-5 and MSRA-7 questionnaires to assess their performance compared to the diagnostic of sarcopenia according to the criteria described by the EWGSOP2, the AWGS-2019 and the IWGS, as well as to other sarcopenia-related outcomes.

### 2.3. Outcome Measures

Demographic data, such as age, education, marital status, occupation, smoking habits, osteoporosis and falls in the last year, were collected. A fall was defined as “an unexpected event in which the participant came to rest on the ground, floor, or lower level” [28].

#### 2.3.1. MSRA

There are two versions of the MSRA questionnaire: the full form with 7 domains or items (MSRA-7) and the short form with 5 domains or items (MSRA-5) [16]. The seven items of the MSRA-7 are (1) age; (2) hospitalizations in the last year; (3) physical activity level; (4) number of daily meals; (5) consumption of dairy products; (6) consumption of dairy proteins; and (7) weight loss in the last year. The MSRA-5 questionnaire includes all these domains except for numbers 5 and 6. The scores for the MSRA-7 items are 0, 5 or 10 and for the MSRA-5 are 0, 5, 10 or 15, and the total score ranges from 0 to 40 (MSRA-7) and from 0 to 60, where higher scores reflect less risk of sarcopenia. A cutoff value of ≤30 (MSAR-7) and of ≤45 (MSRA-5) indicate risk of sarcopenia.

#### 2.3.2. Anthropometrics

Height and weight were measured using an adult height scale (T201-T4 Asimed, Barcelona, Spain) and a precision digital weight scale (Tefal, Barcelona, Spain) with a range of 100 g to 130 kg, respectively. Body mass index (BMI) was calculated using the following formula: BMI (kg/m^2^) = body weight/height^2^ [29].

#### 2.3.3. Muscle Strength 

Muscle strength was assessed using a handgrip dynamometer (TKK 5001, Grip-A, Takei, Tokyo, Japan). Low muscle strength was determined using the handgrip strength cutoff scores of <16 kg (women) and <27 kg (men) according to the EWGSOP2 [1] and <18 kg (women) and <28 kg (men) according to the AWGS-2019 [6]. The IWGS does not include muscle strength in the diagnosis of sarcopenia. 

#### 2.3.4. Muscle Mass 

Muscle mass was evaluated using bioelectrical impedance analysis (BIA) with the InBody 720 device (Biospace Co., Ltd., Seoul, Republic of Korea). The analysis was performed under the same conditions (hydration, exercise, fasting, time of day, etc.) according to the manufacturer’s instructions and recommendations [30]. Appendicular skeletal muscle mass index (ASMI) was determined by dividing the skeletal muscle mass by the square of each participant’s height (kg/m^2^) [31]. A cutoff point for low muscle mass was set at 5.5 kg/m^2^ (women) and 7 kg/m^2^ (men) as described by the EWGSOP2, 5.7 kg/m^2^ (women) and 7 kg/m^2^ (men) in accordance with the AWGS-2019 [6] and 5.67 kg/m^2^ (women) and 7.23 kg/m^2^ (men) according to the IWGS [4]. 

#### 2.3.5. Gait Speed 

The Timed Up-and-Go test was used to evaluate usual gait speed, which was obtained using the following formula: [6/(TUG time) * 1.62] [32]. The cutoff point as described by the EWGSOP2 [1] and the AWGS-2019 [6] is 0.8 m/s and 1 m/s according to the IWGS [4].

#### 2.3.6. Assessment of Sarcopenia

Sarcopenia was diagnosed as low muscle strength and mass (EWGSOP2) [1], low muscle mass and low gait speed (IWGS) [4] and low muscle mass together with low muscle strength or low gait speed (AWGS-2019) [6].

### 2.4. Statistical Analysis

We used SPSS software, version 20.0 (SPSS, Inc., Chicago, IL, USA) for data handling and statistical analysis. An α value ≤ 0.05 indicated statistical significance. To evaluate the normality of the data distribution, the Kolmogorov–Smirnov test was used. Differences between categorical variables (percentages and frequencies) were analyzed using the chi-square test, while Student’s t-tests were applied to compare continuous variables (mean and standard deviation (SD). To assess test–retest and inter-rater reliability, the intraclass correlation coefficient (ICC2,1), as outlined by Shrout and Fleiss, was used. Reliability was categorized as poor with an ICC below 0.40, moderate between 0.40 and 0.75, substantial from 0.75 to 0.90 and excellent with an ICC above 0.90 [33]. As for the clinical validation of the Spanish MSRA questionnaires, specificity (Sp), sensitivity (Se), accuracy, negative predictive value (NPV) and positive predictive value (PPV) were calculated using the diagnostic criteria for sarcopenia described in the EWGSOP2, AWGS-2019 and IWGS. The area under the curve (AUC) was calculated using receiver operating characteristic (ROC) analysis. Lastly, Pearson correlation coefficients were used to examine the relationships between the total scores of the MSRA-5 and the MSRA-7 with muscle strength, ASMI, gait speed and age. 

## 3. Results

The characteristics of all the participants and according to gender (68.38% women) are presented in Table 1. As for the sarcopenia diagnostic parameters, men showed significantly higher values of muscle strength (*p* < 0.001) and muscle mass (*p* = 0.005), but there were no significant differences regarding BMI. With respect to the risk of sarcopenia, men had significantly higher scores in the MSRA-5 total score (*p* = 0.044), but we did not observe significant differences in the MSRA-7 total score. In the rest of the descriptive characteristics, there were no significant differences between groups except for the education level (67.44% of men with secondary or university studies, *p* = 0.008). 

Regarding the answers to the MSRA questionnaire items (Table 2), the majority of the participants (63.24%) were ≥70 years old, not hospitalized in the last year (58.82%), able to walk more than 1000 m, consumed three meals per day regularly (67.65%), consumed dairy products and proteins at least once a day (64.71% and 66.18%, respectively), and 50% lost at least 2 kg in the past year. There were no differences between men and women. 

The prevalence of sarcopenia (Table 3) was similar for the different diagnosis criteria employed and ranged from 8.09% (IWGS) to 13.24% (AWGS-2019), while 69.12% (MSRA-5) and 79.4% (MSRA-7) were at risk of sarcopenia. As for gender, significant differences were observed only in the diagnosis of sarcopenia according the EWGSOP2 (18.60% of men and 5.38% of women, *p* = 0.015), and 55.81% of men and 75.27% of women were at risk of sarcopenia (MSRA-5, *p* = 0.022).

Regarding the inter-rater reliability, our results showed excellent agreement for both the MSRA-5 (ICC = 0.962) and MSRA-7 (ICC = 0.948) questionnaires. With respect to the test–retest reliability, the analysis showed an ICC value of 1 for all the domains except for domain 2 (0.906 and 0.946 for MSRA-5 and MSRA-7, respectively) and total scores (0.988 and 0.997 for the MSRA-5 and the MSRA-7, respectively), which indicates excellent reliability.

Concerning the clinical validation of the Spanish MSRA questionnaires, Table 4 shows the diagnostic value of the questionnaires according to different sarcopenia definitions. Sensitivity values ranged from 88.89% to 94.44% of the AWGS-2019 and from 81.82% to 90.91% of the IWGS (MSRA-5 and MSRA-7, respectively). Specificity values were lower than sensitivity and very similar for the three diagnostic criteria (higher for the AWGS-2019), being greater in case of the MSRA-5 compared to the MSRA-7 (33.90% vs. 22.88%). As for the PPV, the results were low, and again higher values were seen for the AWGS-2019 (17.02% and 15.74% for the MSRA-5 and MSRA-7, respectively), while the NPV values were higher and the same for the three diagnostic criteria in the MSRA-5 (95.24%), while for the MSRA-7, they were 96.30% (IWGS) and 96.43% (EWGSOP2 and AWGS-2019). The accuracy ranged from 41.18% (AWGS-2019) to 36.03% (IWGS) for the MSRA-5 and from 32.35% (AWGS-2019) to 26.47% (IWGS) for the MSRA-7. Our findings revealed moderate AUC values according to the EWGSOP2 for the MSRA-5 (0.755, *p* = 0.003) and MSRA-7 (0.729, *p* = 0.007). Nevertheless, the values were considered low for the AWGS-2019 (0.683, *p* = 0.013 and 0.671, *p* = 0.019 for the MSRA-5 and MSRA-7, respectively) and for the IWGS (0.672, *p* = 0.060 and 0.645, *p* = 0.111 for the MSRA-5 and the MSRA-7, respectively). Finally, Table 5 indicates significant correlations between higher total scores on the MSRA questionnaires and greater handgrip strength, muscle mass and gait speed, as well as with lower age.

## 4. Discussion

The main goal of this study was to carry out the cross-cultural adaptation of the Spanish versions of the MSRA-5 and MSRA-7 questionnaires and to assess their clinical validity in Spanish adults aged 65 years and older. Our results show that the Spanish MSRA questionnaire is a reliable and valid scale for the screening of sarcopenia in Spanish community-dwelling adults aged 65 years and older.

The aging of the population is a global phenomenon, and it is estimated that by 2030, one out of every six people in the world will be ≥60 years old [34]; therefore, the diagnosis and prevention of age-related diseases are very important. As mentioned, sarcopenia has been linked to several adverse outcomes, and it is very important to have tools that facilitate the detection of people who may be at risk of suffering from it, such as the MSRA questionnaire in its five- and seven-item versions. Despite the importance of sarcopenia, there is a lack of unity in the diagnosis of sarcopenia, not only with respect to the operational criteria but also cutoff points and measurement instruments [35], and this represents one of the main obstacles in sarcopenia research, for prevalence studies and in clinical practice [36]. Due to this lack of consensus, we performed the clinical validity of the MSRA according to the criteria of different study groups. In this work, and following the EWGSOP2 criteria, the prevalence of sarcopenia was 9.56%, similar to the 11% described in a recent systematic review published in 2022 by Fernandes et al. [37]. In a study carried out in 2019 in different communities and districts of Beijing (China) [38], the prevalence of sarcopenia according to the AWGS-2019 criteria was higher than that obtained with EWGSOP2, as was observed in the present study, although the percentages observed were lower (8.6% for AWGS-2019 and IWGS and 5.4% for EWGSOP2). Regarding differences by sex, the results of the present study partially coincide with what was described by Petterman-Rocha et al. (2022) in a recent meta-analysis aimed at determining the prevalence of sarcopenia and severe sarcopenia by sociodemographic factors, in which men had a higher prevalence according to the EWGSOP2 criteria, while the percentage was greater in women when following the IWGS criteria [39]. Our findings showed that the prevalence of sarcopenia was higher in men regardless of the criteria, but only significant differences were observed when the EWGSOP2 was used. When analyzing the differences regarding gender, our findings showed higher values (thus lower risk) on both MSRA questionnaires in men, which is in accordance with previous studies [19,22], and, as expected, men also had significantly higher values for muscle mass and strength [23]. However, it should be noted that the number of men who participated in this study was lower than the women (43 vs. 93), and this should be considered when interpreting these gender-related differences.

The test–retest reliability assesses the reproducibility of the scale and indicates how consistent the scores of this tool are over time. In order to determine it, a subsample of 25 participants (50% women) completed the Spanish versions of the MSRA again two weeks later. The number of participants and the time interval was chosen according to previous validations and recommendations [23,24]. Our findings showed excellent reliability, where all the ICC values (both items and total scores) were greater than 0.90. These results are similar than those described by Krzymińska-Siemaszko et al. (2021) [23] and Pantouvaki et al. (2023) [24] in the Polish and Greek versions, respectively. In order to assess the inter-rater reliability, our analysis revealed excellent agreement for the total scores of the Spanish versions of the MSRA questionnaires, while in the Polish validation, excellent (0.910) and good (0.834) ICC values were described for the MSRA-7 and -5, respectively.

As noted previously, the MSRA has a higher sensitivity than the SARC-F but a lower specificity [20], which suggests a lower ability to rule out false negatives. This low specificity may be related to the established cutoff point. In fact, Rossi et al. [19] stated that, considering the prevalence of sarcopenia and the costs associated with false positives and negatives, the relative cost of the latter could be considered greater than that of false positives, and, therefore, this cutoff point was selected since it favors sensitivity more than specificity. As for the clinical validation, when using a cutoff point of ≤30 (MSRA-5) and ≤45 (MSRA-7) to identify subjects with sarcopenia, the results of the present study revealed that sensitivity and NPV were high under the three diagnostic criteria (the greater values were found for the MSRA-7 and under the AWGS-2019 criteria), while low sensitivity and PPV were observed. We found that both questionnaires had sensitivity values greater than 0.80, regardless of the used criteria, and are useful as a sarcopenia screening tool, which is in accordance with previous validations [21,23]. Rossi et al. (2017) in the validation of the original version described a sensitivity of 0.804 for both MRSA questionnaires, although only EWGSOP1 criteria were used [19]. On the other hand, Akarapornkrailert et al. (2020) found, in the Thai validation performed against the AWGS 2019 definition, a sensitivity of 72.3% (MSRA-7) and 61.5% (MSRA-5), although specificity values were higher than ours (43% and 67.4%, respectively) [22]. Our results also showed that, under all the sarcopenia definitions, the MSRA-5 had higher specificity and accuracy than the MSRA- 7, which is in line with the characteristics of the original version of the MSRA [19], as with the Chinese and Polish validations [21,23]. Although the negative predictive values were high, the positive predictive values were low for all criteria, which is in agreement with previous evaluations [22,23]. The low percentage of participants with sarcopenia may be related since as the prevalence decreases, the PPV decreases while the NPV increases [40]. On the other hand, the AUC values were in line with previous validations when using the EWGSOP2 [22,23], the AWGS-2019 [23] and the IWGS operational definitions of sarcopenia [21,23], and we also agree with these authors in finding that the values of the five-item version were higher than those of the seven-item version.

Sarcopenia has been traditionally defined as an age-related muscle mass decline, although for the EWGSOP2, muscle strength is the primary parameter of sarcopenia [1] since it is better than muscle mass in predicting poor patient outcomes [41,42], and lower handgrip strength has been associated with higher all-cause, cancer and cardiovascular mortality risk [43]. Physical performance is the third parameter used in the diagnostic criteria of sarcopenia, often assessed by gait speed, which has been related to disability, frailty, lower quality of life and mortality in community-dwelling older adults [44]. In this study, we found that higher MSRA total scores (and therefore lower risk of sarcopenia) were associated, as well as older age with higher values in these three sarcopenia-related parameters, although the r values were low, which is in line with the results described by Krzymińska-Siemaszko et al. (2021) in the Polish validation [23]. This may be related to the fact that the MSRA questionnaires consider other sarcopenia risk factors than physical fitness, such as regular meals, adequate protein consumption, weight loss and hospitalizations. 

There are some limitations of the present study that should be considered. It was carried out in a sample population of 136 community-dwelling older adults in which the number of men can be considered too small (31.62%) to produce statistically reliable results when interpreting descriptive gender–related differences. Moreover, the participants belong to one specific region of Spain, and, thus, any generalization of our results should be made with caution, and more studies should be completed in future in a more general sample population with a balanced proportion between men and women from various geographical areas. In addition, the use of a bioelectrical impedance analysis method to evaluate muscle mass is not the most precise method as compared with dual-energy X-ray absorptiometry, magnetic resonance imaging or computed tomography, even though they are not commonly employed given the high costs, the lack of portability and the need for qualified personnel [45]. Finally, although the number of participants can be considered as acceptable for this study, future studies conducted on a greater sample size with a balanced ratio between men and women would add robustness to the results.

## 5. Conclusions

The MSRA-5 and MSRA-7 questionnaires were cross-culturally adapted and validated in community-dwelling Spanish older adults. The analysis showed excellent inter-rater and test–retest reliability. The specificity and negative predictive values make the MSRA questionnaires an appropriate tool for sarcopenia screening in community-dwelling older adults under different sets of diagnostic criteria. The accuracy and the area under the curve were better for the MSRA-5 as compared with the MSRA-7, and higher MSRA-5 and MSRA-7 total scores significantly correlated with greater muscle strength, quantity and gait speed.

## Figures and Tables

**Table 1:** Characteristics of all the participants and according to gender.

		Total (n = 136)		Men (n = 43)		Women (n = 93)		*p*-Value
Age ^a^		72.24	5.21	71.86	5.15	72.41	5.25	0.570
Occupation ^b^	Retired	116	85.29	39	90.70	77	82.80	0.416
	Active worker	3	2.21	1	2.33	2	2.15	
	Unemployed	17	12.50	3	6.98	14	15.05	
Marital status ^b^	Single	30	22.06	9	20.93	21	22.58	0.287
	Married	70	51.47	26	60.47	44	47.31	
	Separated/divorced/widowed	36	26.47	8	18.60	28	30.11	
Education ^b^	Primary or less	67	49.3	14	32.56	53	56.99	0.008 *
	Secondary or higher	69	50.7	29	67.44	40	43.01	
Falls in the last year ^b^	No	72	52.94	22	51.16	50	53.76	0.778
	Yes	64	47.06	21	48.84	43	46.24	
BMI ^a^		27.77	3.46	27.38	3.45	27.95	3.47	0.367
Handgrip strength ^a^		19.67	6.89	26.72	7.09	16.41	3.57	<0.001 *
ASMI ^a^		7.15	1.95	7.83	1.68	6.83	2.00	0.005 *
Gait speed ^a^		1.12	0.28	1.15	0.29	1.11	0.27	0.391
MSRA7 total score ^a^		25.04	7.56	26.86	7.32	24.19	7.56	0.056
MSRA5 total score ^a^		38.68	13.50	42.09	14.28	37.10	12.90	0.044 *

BMI: body mass index. MSRA: Mini Sarcopenia Risk Assessment. ASMI: Appendicular skeletal muscle mass index. ^a^ Values expressed as means and standard deviations. ^b^ Values expressed as frequencies and percentages. * *p* value < 0.05.

**Table 2:** Results of the MSRA items in general and classified by gender.

		All (n = 136)		Men (n = 43)		Women (n = 93)		*p*-Value
Q1. Age	≥70 years	86	63.24	22	51.16	64	68.82	0.057
	<70 years	50	36.76	21	48.84	29	31.18	
Q2. Number of hospital treatments in the last year	Yes, more than once	8	5.88	3	6.98	5	5.38	0.467
	Yes, once	48	35.29	12	27.91	36	38.71	
	No	80	58.82	28	65.12	52	55.91	
Q3. Level of physical activity	Able to walk < 1000 m	51	37.50	13	30.23	38	40.86	0.234
	Able to walk > 1000 m	85	62.50	30	69.77	55	59.14	
Q4. Regular consumption of three meals a day	No, up to twice a week, I skip a meal	44	32.35	12	27.91	32	34.41	0.451
	Yes	92	67.65	31	72.09	61	65.59	
Q5. Consumption of dairy products	Not every day	48	35.29	12	27.91	36	38.71	0.220
	At least once a day	88	64.71	31	72.09	57	61.29	
Q6. Consumption of proteins	Not every day	46	33.82	18	41.86	28	30.11	0.178
	At least once a day	90	66.18	25	58.14	65	69.89	
Q7. Weight loss in the last year	>2 kg	68	50.00	18	41.86	50	53.76	0.197
	≤2 kg	68	50.00	25	58.14	43	46.24	

Values expressed as frequencies and percentages. MSRA: Mini Sarcopenia Risk Assessment.

**Table 3:** Prevalence of sarcopenia according to different diagnosis criteria and gender.

Sarcopenia Diagnosis		Total (n = 136)		Men (n = 43)		Women (n = 93)		*p*-Value
EWGSOP2	No sarcopenia	123	90.44	35	81.40	88	94.62	0.015 *
	Sarcopenia	13	9.56	8	18.60	5	5.38	
AWGS-2019	No sarcopenia	118	86.76	35	81.40	83	89.25	0.209
	Sarcopenia	18	13.24	8	18.60	10	10.75	
IWGS	No sarcopenia	125	91.91	37	86.05	88	94.62	0.088
	Sarcopenia	11	8.09	6	13.95	5	5.38	
MSRA-5	No risk of sarcopenia	42	30.88	19	44.19	23	24.73	0.022 *
	Risk of sarcopenia	94	69.12	24	55.81	70	75.27	
MSRA-7	No risk of sarcopenia	28	20.59	13	30.23	15	16.13	0.059
	Risk of sarcopenia	108	79.41	30	69.77	78	83.87	

Values expressed as frequencies and percentages. AWGS: Asian Working Group on Sarcopenia. EWGSOP2: Revised European Working Group of Sarcopenia in Older People. IWGS: International Working Group on Sarcopenia. MSRA: Mini Sarcopenia Risk Assessment. * *p* value < 0.05.

**Table 4:** Diagnostic values of the MSRA-5 and MSRA-7 with respect to different sarcopenia operational criteria.

	Risk of Sarcopenia							
		Se	Sp	PPV	NPV	Acc	AUC (95% CI)	*p*-Value
EWGSOP2	MSRA-5	84.62	32.52	11.70	95.24	37.50	0.76 (0.64–0.87)	0.003 *
	MSRA-7	92.31	21.95	11.11	96.43	28.68	0.73 (0.61–0.85)	0.007 *
AWGS-2019	MSRA-5	88.89	33.90	17.02	95.24	41.18	0.68 (0.58–0.79)	0.013 *
	MSRA-7	94.44	22.88	15.74	96.43	32.35	0.67 (0.55–0.79)	0.019 *
IWGS	MSRA-5	81.82	32.00	9.57	95.24	36.03	0.67 (0.54–0.80)	0.060
	MSRA-7	90.91	20.80	9.17	96.30	26.47	0.65 (0.50–0.79)	0.111

Values expressed as percentages. Acc: accuracy. AUC: area under the receiver operating characteristic curve. AWGS: Asian Working Group on Sarcopenia. CI: confidence interval. EWGSOP2: Revised European Working Group of Sarcopenia in Older People. IWGS: International Working Group on Sarcopenia. MSRA: Mini Sarcopenia Risk Assessment. PPV: positive predictive value. NPV: negative predictive value. Se: sensitivity. Sp: specificity. * *p* value < 0.05.

**Table 5:** Correlation between the total scores of the MSRA questionnaires and other related variables.

	MSRA-5		MSRA-7	
	r	*p*-Value	r	*p*-Value
Age	−0.577	<0.001	−0.536	<0.001 *
Handgrip strength	0.292	0.001	0.338	<0.001 *
ASMI	0.212	0.013	0.172	0.046 *
Gait speed	0.172	0.045	0.179	0.037 *

MSRA: Mini Sarcopenia Risk Assessment. r: Pearson correlation coefficient. ASMI: skeletal muscle mass index. * *p* value < 0.05.

## Data Availability

The original contributions presented in the study are included in the article, further inquiries can be directed to the corresponding author/s.

## References

[1] Cruz-Jentoft A.J., Bahat G., Bauer J., Boirie Y., Bruyère O., Cederholm T., Cooper C., Landi F., Rolland Y., Sayer A.A. (2019). Sarcopenia: Revised European consensus on definition and diagnosis. Age Ageing.

[2] Rosenberg I. (1989). Summary comments: Epidemiological and methodological problems in determining nutritional status of older persons. Am. J. Clin. Nutr..

[3] Cruz-Jentoft A.J., Baeyens J.P., Bauer J.M., Boirie Y., Cederholm T., Landi F., Martin F.C., Michel J.P., Rolland Y., Schneider S.M. (2010). Sarcopenia: European consensus on definition and diagnosis: Report of the European Working Group on Sarcopenia in Older People. Age Ageing.

[4] Fielding R.A., Vellas B., Evans W.J., Bhasin S., Morley J.E., Newman A.B., Abellan van Kan G., Andrieu S., Bauer J., Breuille D. (2011). Sarcopenia: An undiagnosed condition in older adults. Current consensus definition: Prevalence, etiology, and consequences. International working group on sarcopenia. J. Am. Med. Dir. Assoc..

[5] Chen L.K., Liu L.K., Woo J., Assantachai P., Auyeung T.W., Bahyah K.S., Chou M.Y., Chen L.Y., Hsu P.S., Krairit O. (2014). Sarcopenia in Asia: Consensus report of the Asian Working Group for Sarcopenia. J. Am. Med. Dir. Assoc..

[6] Chen L.K., Woo J., Assantachai P., Auyeung T.W., Chou M.Y., Iijima K., Jang H.C., Kang L., Kim M., Kim S. (2020). Asian Working Group for Sarcopenia: 2019 Consensus Update on Sarcopenia Diagnosis and Treatment. J. Am. Med. Dir. Assoc..

[7] Anker S.D., Morley J.E., von Haehling S. (2016). Welcome to the ICD-10 code for sarcopenia. J. Cachexia Sarcopenia Muscle.

[8] Rivera F.B., Escolano B.T., Nifas F.M., Choi S., Carado G.P., Lerma E., Vijayaraghavan K., Yu M.G. (2024). Interrelationship of Sarcopenia and Cardiovascular Diseases: A Review of Potential Mechanisms and Management. J. ASEAN Fed. Endocr. Soc..

[9] Amini N., Ibn Hach M., Lapauw L., Dupont J., Vercauteren L., Verschueren S., Tournoy J., Gielen E. (2024). Meta-analysis on the interrelationship between sarcopenia and mild cognitive impairment, Alzheimer’s disease and other forms of dementia. J. Cachexia Sarcopenia Muscle.

[10] Fatani H., Olaru A., Stevenson R., Alharazi W., Jafer A., Atherton P., Brook M., Moran G. (2023). Systematic review of sarcopenia in inflammatory bowel disease. Clin. Nutr..

[11] Lin T.Y., Chen Y.F., Wu W.T., Han D.S., Tsai I.C., Chang K.V., Özçakar L. (2022). Impact of sarcopenia on the prognosis and treatment of lung cancer: An umbrella review. Discov. Oncol..

[12] Giakoustidis A., Papakonstantinou M., Chatzikomnitsa P., Gkaitatzi A.D., Bangeas P., Loufopoulos P.D., Louri E., Myriskou A., Moschos I., Antoniadis D. (2024). The Effects of Sarcopenia on Overall Survival and Postoperative Complications of Patients Undergoing Hepatic Resection for Primary or Metastatic Liver Cancer: A Systematic Review and Meta-Analysis. J. Clin. Med..

[13] Lin W.L., Nguyen T.H., Huang W.T., Guo H.R., Wu L.M. (2024). Sarcopenia and survival in colorectal cancer without distant metastasis: A systematic review and meta-analysis. J. Gastroenterol. Hepatol..

[14] Beaudart C., Demonceau C., Reginster J.Y., Locquet M., Cesari M., Cruz Jentoft A.J., Bruyère O. (2023). Sarcopenia and health-related quality of life: A systematic review and meta-analysis. J. Cachexia Sarcopenia Muscle.

[15] De Buyser S.L., Petrovic M., Taes Y.E., Toye K.R., Kaufman J.M., Lapauw B., Goemaere S. (2016). Validation of the FNIH sarcopenia criteria and SOF frailty index as predictors of long-term mortality in ambulatory older men. Age Ageing.

[16] Mijnarends D.M., Luiking Y.C., Halfens R.J.G., Evers S.M.A.A., Lenaerts E.L.A., Verlaan S., Wallace M., Schols J.M.G.A., Meijers J.M.M. (2018). Muscle, Health and Costs: A Glance at their Relationship. J. Nutr. Health Aging.

[17] Malmstrom T.K., Morley J.E. (2013). SARC-F: A simple questionnaire to rapidly diagnose sarcopenia. J. Am. Med. Dir. Assoc..

[18] Ida S., Kaneko R., Murata K. (2018). SARC-F for Screening of Sarcopenia Among Older Adults: A Meta-analysis of Screening TestAccuracy. J. Am. Med. Dir. Assoc..

[19] Rossi A.P., Micciolo R., Rubele S., Fantin F., Caliari C., Zoico E., Mazzali G., Ferrari E., Volpato S., Zamboni M. (2017). Assessing the Risk of Sarcopenia in the Elderly: The Mini Sarcopenia Risk Assessment (MSRA) Questionnaire. J. Nutr. Health Aging.

[20] Yang M., Hu X., Xie L., Zhang L., Zhou J., Lin J., Wang Y., Li Y., Han Z., Zhang D. (2019). Comparing Mini Sarcopenia Risk Assessment With SARC-F for Screening Sarcopenia in Community-Dwelling Older Adults. J. Am. Med. Dir. Assoc..

[21] Yang M., Hu X., Xie L., Zhang L., Zhou J., Lin J., Wang Y., Li Y., Han Z., Zhang D. (2018). Validation of the Chinese version of the Mini Sarcopenia Risk Assessment questionnaire in community-dwelling older adults. Medicine.

[22] Akarapornkrailert P., Muangpaisan W., Boonpeng A., Daengdee D. (2020). Validation of the Thai version of SARC-F, MSRA-7, and MSRA-5 questionnaires compared to AWGS 2019 and sarcopenia risks in older patients at a medical outpatient clinic. Osteoporos. Sarcopenia.

[23] Krzymińska-Siemaszko R., Deskur-Śmielecka E., Styszyński A., Wieczorowska-Tobis K. (2021). Polish Translation and Validation of the Mini Sarcopenia Risk Assessment (MSRA) Questionnaire to Assess Nutritional and Non-Nutritional Risk Factors of Sarcopenia in Older Adults. Nutrients.

[24] Ribeiro L.S., Souza B.G.A., de Lima J.B., Pimentel G.D. (2021). Cross-Cultural Adaptation of the Brazilian Portuguese-Translated Version of the Mini Sarcopenia Risk Assessment (MSRA) Questionnaire in Cancer Patients. Clin. Pract..

[25] Pantouvaki A., Kastanis G., Patelarou E., Alpantaki K., Kleisiaris C., Zografakis-Sfakianakis M. (2023). Greek Translation, Cultural Adaptation and Validation of the Mini Sarcopenia Risk Assessment Questionnaire, to Evaluate Sarcopenia in Greek Elderly at a Hospital Setting. Nurs. Rep..

[26] Kline P. (1993). An Easy Guide to Factor Analysis.

[27] World Health Organization (2023). Adaptation and Translation Guide.

[28] Lamb S.E., Jørstad-Stein E.C., Hauer K., Becker C. (2005). Prevention of Falls Network Europe and Outcomes Consensus Group. Development of a common outcome data set for fall injury prevention trials: The Prevention of Falls Network Europe consensus. J. Am. Geriatr. Soc..

[29] World Health Organization (2000). Obesity: Preventing and Management of the Global Epidemic.

[30] InBody 720 User’s Manual. https://www.inbody.in/uploads/resource/inbody720_cdmanual_eng_h-pdf-0059383001546245174.pdf.

[31] Chien M.Y., Huang T.Y., Wu Y.T. (2008). Prevalence of sarcopenia estimated using a bioelectrical impedance analysis prediction equation in community-dwelling elderly people in Taiwan. J. Am. Geriatr. Soc..

[32] Lauretani F., Russo C.R., Bandinelli S., Bartali B., Cavazzini C., Di Iorio A., Corsi A.M., Rantanen T., Guralnik J.M., Ferrucci L. (2003). Age-associated changes in skeletal muscles and their effect on mobility: An operational diagnosis of sarcopenia. J. Appl. Physiol..

[33] Shrout P.E., Fleiss J.L. (1979). Intraclass correlations: Uses in assessing rater reliability. Psychol. Bull..

[34] World Health Organization Ageing and Health. https://www.who.int/es/news-room/fact-sheets/detail/ageing-and-health.

[35] Stuck A.K., Tsai L.T., Freystaetter G., Vellas B., Kanis J.A., Rizzoli R., Kressig K.S., Armbrecht G., Da Silva J.A.P., Dawson-Hughes B. (2023). Comparing Prevalence of Sarcopenia Using Twelve Sarcopenia Definitions in a Large Multinational European Population of Community-Dwelling Older Adults. J. Nutr. Health Aging.

[36] Boshnjaku A., Krasniqi E. (2024). Diagnosing sarcopenia in clinical practice: International guidelines vs. population-specific cutoff criteria. Front. Med..

[37] Fernandes L.V., Paiva A.E.G., Silva A.C.B., de Castro I.C., Santiago A.F., de Oliveira E.P., Porto L.C.J. (2022). Prevalence of sarcopenia according to EWGSOP1 and EWGSOP2 in older adults and their associations with unfavorable health outcomes: A systematic review. Aging Clin. Exp. Res..

[38] Cao M., Lian J., Lin X., Liu J., Chen C., Xu S., Ma S., Wang F., Zhang N., Qi X. (2022). Prevalence of sarcopenia under different diagnostic criteria and the changes in muscle mass, muscle strength, and physical function with age in Chinese old adults. BMC Geriatr..

[39] Petermann-Rocha F., Balntzi V., Gray S.R., Lara J., Ho F.K., Pell J.P., Celis-Morales C. (2022). Global prevalence of sarcopenia and severe sarcopenia: A systematic review and meta-analysis. J. Cachexia Sarcopenia Muscle.

[40] Tenny S., Hoffman M.R. (2023). Prevalence. StatPearls [Internet].

[41] Leong D.P., Teo K.K., Rangarajan S., Lopez-Jaramillo P., Avezum A., Orlandini A., Seron P., Ahmed S.H., Rosengren A., Kelishadi R. (2015). Prognostic value of grip strength: Findings from the Prospective Urban Rural Epidemiology (PURE) study. Lancet.

[42] Wang Y., Pu X., Zhu Z., Sun W., Xue L., Ye J. (2023). Handgrip strength and the prognosis of patients with heart failure: A meta-analysis. Clin. Cardiol..

[43] López-Bueno R., Andersen L.L., Koyanagi A., Núñez-Cortés R., Calatayud J., Casaña J., Del Pozo Cruz B. (2022). Thresholds of handgrip strength for all-cause, cancer, and cardiovascular mortality: A systematic review with dose-response meta-analysis. Ageing Res. Rev..

[44] Binotto M.A., Lenardt M.H., Rodríguez-Martínez M.D.C. (2018). Physical frailty and gait speed in community elderly: A systematic review. Rev. Esc. Enferm. USP.

[45] Beaudart C., McCloskey E., Bruyère O., Cesari M., Rolland Y., Rizzoli R., de Araujo Carvalho I., Amuthavalli Thiyagarajan J., Bautmans I., Bertière M.C. (2016). Sarcopenia in daily practice: Assessment and management. BMC Geriatr..

